# Addition of Chinese medicinal herbal residues promotes the removal of antibiotic resistance genes during pig manure composting

**DOI:** 10.3389/fmicb.2026.1932472

**Published:** 2026-10-14

**Authors:** Bo Wang, Lanlan Tian, Yibo Wang, Zexing Tian, Yanrong Yang

**Affiliations:** 1 College of Bioengineering and Biotechnology, Tianshui Normal University, Tianshui, China; 2 Gansu Provincial Key Laboratory for Utilization of Agricultural Solid Waste Resources, Tianshui, China

**Keywords:** aerobic composting, antibiotic resistance genes, Chinese medicinal herbal residues, mobile genes elements, pig manure

## Abstract

Aerobic composting is an effective methods for reducing antibiotic resistance genes (ARGs) and mobile genetic elements (MGEs) present in livestock and poultry manure. Chinese medicinal herbal residues (CMHRs) are rich in nutrients and antibacterial components, which may inhibit the spread of ARGs and MGEs. This study investigated the effects of adding different proportions of CMHRs added (0%, 10%, 30%, and 50% of the initial compost mixture mass) on ARGs and MGEs during pig manure composting. The results showed that CMHRs shortened the time required to reach the high-temperature stage and increased compost temperature. At the end of composting, the total nitrogen (TN) and available phosphorus (AP) levels in the M2 treatment group were significantly higher than those in the control group, reaching 8.11 g/kg and 333.73 mg/kg, respectively. High-throughput qPCR analysis showed that the total removal rates of ARGs after composting were 27.10%, 36.04%, 65.55%, and 40.55%, respectively; the corresponding removal rates of MGEs were 19.25%, 56.20%, 65.32%, and 14.03%, respectively; The 30% treatment was the most effective, with removal rates of aminoglycosides, β-lactam, multidrug resistance, sulfonamides, and tetracycline resistance genes of 78.09%, 81.11%, 74.09%, 54.27%, and 61.24%, respectively. Firmicutes, Proteobacteria, Actinobacteria, and Bacteroidetes were the dominant phyla in the compost. *Clostridium-sensu-stricto1*, *Corynebacterium*, *Terrisporobacter*, *Anaerococcus*, *and Romboutsia* were potential major hosts of ARGs and MGEs. During composting, changes in temperature and MGEs directly and significantly affected ARGs, while total organic carbon (TOM) and TN indirectly affected ARGs by regulating microbial communities. In conclusion, the addition of 30% CMHRs effectively reduces ARGs and MGEs in pig manure compost. These findings provide a basis for the use of CMHRs to reduce the spread of ARGs during pig manure composting.

## 1 Introduction

With the rapid development of the global livestock and poultry industry, animal manure production has sharply increased; however, its effective treatment and comprehensive utilisation have often been overlooked. For example, the annual production of animal manure in China has exceeded 4 billion tons, whereas its comprehensive utilisation rate remains below 60% (Qi et al., 2023; Wu H. et al., 2020). Livestock manure, which is rich in nutrients and organic matter, is an important material for the production of organic agricultural fertilisers (Wang et al., 2016). To prevent diseases and promote the growth of livestock and poultry, antibiotics are widely used as feed additives. However, 50–80% of antibiotics are directly excreted directly by livestock. These antibiotics can exert selective pressure in the environment, leading to the emergence and accumulation of antibiotic resistance genes (ARGs) (Zhang et al., 2024). Because ARGs can be horizontally transferred via mobile genetic elements (MGEs), they ultimately pose a serious threat to the environment and human health (Xu et al., 2024).

One of the most effective methods for recovering resources from livestock and poultry manure and reducing its potential adverse effects is aerobic composting (Ren et al., 2019). Many studies have shown that composting is an important method for reducing antibiotic concentrations and the abundance of antibiotic resistance genes (Huang et al., 2021; Ma et al., 2023; Wang B. et al., 2024; Wang et al., 2024a). Composting parameters, such as temperature, pH, and turning frequency, can significantly affect the composting process, the degree of product humification, and the reduction of ARGs (Cai et al., 2025; Wang et al., 2015; Wu N. et al., 2020; Yu et al., 2023). Furthermore, recent research has indicated that exogenous additives can further enhance ARGs removal during composting can further enhance the removal rate of ARGs (Cheng et al., 2025a). For example, in a study of aerobic composting of cow manure, the addition of different proportions of straw altered the microbial community structure and thereby affected ARGs abundance. The results also suggested that *Bacillus* may have been particularly important in controlling ARGs abundance (Cao et al., 2025). Qiu et al. (2021) added high, medium, and low-quality substrates to compost containing 10% chicken manure. The results showed that the levels of *tetW*, *ermC*, *sul1*, and *sul2* level were lowest in the group treated with high-quality substrate. Changes in physicochemical properties were considered the main factors contributing to the decrease in ARG levels. In trials of aerobic sheep manure composting, the addition of maize straw and apple branches increased the levels of total nitrogen (TN), total phosphorus, and total potassium. A comprehensive analysis suggested that the addition of maize straw effectively mitigated the risk of ARG proliferation and transmission (Qin et al., 2024).

Biochar has been widely used as a composting additive due to its porous, hydrophobic, and low-density characteristics. Numerous studies have shown that biochar promotes the degradation of organic matter and significantly reduces ARG abundance during composting by altering microbial community structure (Chen et al., 2022; Li et al., 2023; Liu H. et al., 2021; Zhou et al., 2022). In addition, incorporating novel additives, such as functional microorganisms and nano-zero-valent iron (Qiu X. et al., 2022; Yan et al., 2025) into the composting process can enhance microbial activity and metabolism, alter microbial community succession, modulate physicochemical properties, and indirectly reduce ARG abundance, ultimately improving compost quality.

In recent years, particularly during the coronavirus disease pandemic, the use of Chinese medicinal herbs residues (CMHRs) increased, resulting in the generation of substantial quantities of CMHRs (Wang et al., 2021). Statistics indicate that China produces approximately 70 million tons of CMHRs annually (Hou et al., 2022). If these residues are not properly managed, substantial resource waste and environmental pollution may result (Lu and Li, 2020). CMHRs are rich in components such as cellulose, polysaccharides, amino acids, proteins, and alkaloids. In addition, they contain substantial amounts of nutrients such as nitrogen, phosphorus, and potassium, as well as biologically active compounds (Liu R. et al., 2021). The surface of CMHRs contains numerous pores and functional groups, suggesting that these residues may have considerable adsorption capacity (Qiu Z. et al., 2022). Previous studies have shown that biochar prepared from CMHRs effectively adsorbs tetracycline (Zhao Z. et al., 2024), norfloxacin (Li et al., 2024), phosphate and ammonium (Wang et al., 2024b) from wastewater.

In addition to using the adsorption properties of CMHR-derived biochar to purify wastewater treatment, the use of raw CMHRs as exogenous additives has received increasing attention in the field of aerobic composting. A previous study showed that co-composting chicken manure with CMHRs as an auxiliary agent significantly altered the bacterial community structure. Changes in abundance of bacteria considered potential hosts of ARGs were closely associated with changes in ARG levels (Guo et al., 2022). Zhou et al., (2018) also reported that using CMHRs as additives to regulate the C/N ratio during kitchen waste composting significantly improved organic matter degradation and seed germination rate. These studies indicate the potential of CMHRs to improve composting and reduce ARGs. However, relatively few studies have investigated changes in ARGs and MGEs during livestock and poultry manure composting with CMHRs. In particular, the relationships among microbial communities, changes in ARGs, and the environmental factors remains unclear. Therefore, in this study, different proportions of CMHRs were added to a pig manure composting system, and their effects on compost physicochemical properties, ARGs, MGEs, and microbial community were systematically investigated.

This study aimed to elucidate the microbial mechanisms underlying ARGs dynamics and the primary factors effected ARG variation during the co-composting of CMHRs and pig manure. The findings may provide a theoretical basis for reducing the environmental risks associated with ARGs in compost products and promoting the sustainable recycling of livestock and poultry manure.

## 2 Materials and methods

### 2.1 Composting experimental design and sample collection

The pig manure and CMHRs used in the composting experiment were obtained from Fengyuan Breeding Farmers Professional Cooperative and Qihuang Pharmaceutical Co., Ltd., respectively, in Tianshui, Gansu, China. The main components of the CMHRs included Cistanches Herba and Aurantii Fructus Immaturus. The material was dried and crushed into fragments below 2 cm for future use. Composting experiment was conducted at the experimental base of Tianshui Normal University. Pig manure was mixed with corn stover and the initial C/N ratio of the compost was adjusted to about 25. Supplementary Tables S1, S2, respectively, display the physical and chemical properties of the compost raw materials and basic information about the tested CMHRs. In this experiment, the amount of CMHRs added was optimised based on previous research (Guo et al., 2022). CMHRs (dry weight) were added at 10%, 30%, and 50% of the initial compost mixture mass (dry weight), with the following groups: CK (pig manure and corn stover), M1 (pig manure, corn stover, and 10% CMHRs), M2 (pig manure, corn stover, and 30% CMHRs), and M3 (pig manure, corn stover, and 50% CMHRs). All treatments were repeated three times. The materials were evenly mixed and placed in a 115-litre foam box, and sterile water was added to adjust the moisture content to about 60%. No extra water was added during the composting process. Two 2 cm × 2 cm holes were made on each side of the foam box, and the compost was turned manually every 5 days to maintain suitable aerobic conditions.

The entire composting process lasted for 40 days. Compost samples were collected on days 0, 5, 25, and 40. To obtain homogenised material, samples were collected from the upper, middle, and lower parts of the compost container and mixed thoroughly (Qiu X. et al., 2022). The collected samples were divided into two portions, with one portion stored in a 4 °C refrigerator for determining physical and chemical indicators, and another one stored in a −80 °C for determining ARGs and microbial communities.

### 2.2 Analysis of the compost physical and chemical indicators

The compost temperature was measured at different positions of the composting device (upper, middle, and lower). The total organic matter (TOM), total nitrogen (TN), and available phosphorus (AP) content were determined using potassium dichromate volumetric method, Kjeldahl nitrogen determination method, and sodium bicarbonate leaching molybdenum antimony colorimetric method, respectively. PH and conductivity were measured using potentiometric and electrode methods, respectively (Chen et al., 2019).

### 2.3 DNA extraction and high-throughput qPCR

Total DNA was extracted from a 0.1 g compost sample using a fast DNA extraction kit for soil (MP Biomedical, USA) reagent kit. The quality and concentration of the DNA were determined using 1% agarose gel electrophoresis and a Nanodrop ND-1000 spectrophotometer (Thermo Fisher Scientific, USA), respectively.

High throughput qPCR was performed using a SmartChip real-time PCR system from Guangdong Magi Gene Technology Co., Ltd. A total of 144 sets of primers were used (primers are listed in Supplementary Table S3), including primers for 36 aminoglycoside resistance genes, 13 β-lactam resistance genes, 7 diaminopyrimidine resistance genes, 6 fluoroquinolone resistance genes, 7 glycopeptide resistance genes, 16 macrolide–lincosamide–streptogramin B (MLSB) resistance genes, 16 multidrug resistance genes, 2 peptide resistance genes, 7 phenol resistance genes, 3 sulfonamide resistance genes, 17 tetracyclines, 6 MGEs, 2 insertional sequences, 1 integrase, 2 plasmids, 2 transposases, and 1 16S rRNA. qPCR was performed in a 100-nL reaction system with 1 × Light Cycler 480 SYBR Green I Master, 500 nM primer (each), and 2 ng·μL − 1 DNA template. After the initial enzyme activation at 95 °C for 10 min, the following amplification conditions were used: denaturation at 95 °C for 30 s and annealing at 60 °C for 30 s. The melting process was automatically generated by Wafergen software. After the reaction was completed, the results were analysed using SmartChip qPCR software to exclude wells with multiple melting peaks or amplification efficiencies exceeding the specified range (90–110%). The results of the remaining wells were then screened using the following criteria: (1) threshold cycle (CT) < 31 and (2) positive samples were indicated in all three replicates. The gene copy number was determined using the following formula: gene copy number = 10exp[(31 − CT) × (10/3)]. The relative abundance of genes was the ratio of the relative copy number of genes to the relative copy number of 16S rRNA. Using a LineGene 9,600 Plus instrument, absolute quantitative information of 16S rRNA was obtained. The absolute abundance of the other genes was then obtained using the following relationship: relative abundance of 16S rRNA/absolute abundance of 16S rRNA = relative abundance of the gene/absolute abundance of the gene (Wang B. et al., 2024).

### 2.4 16S rRNA high-throughput sequencing

The high-throughput sequencing of the 16S rRNA in the microbial communities was performed using an Illumina NovaSeq 6,000 platform at Beijing Baimaike Biotechnology Co., Ltd. (Beijing, China). The 16S rRNA V3–V4 region was selected for amplification sequencing using primers 341F (5′-ACTCCTACGGGAGGCAGCA-3′) and 806R (5′-GGACTACHVGGGTWTCTAAT-3′). Trimomatic v0.33 software was used to filter the raw sequencing data and obtain high-quality sequences. The sequences were then clustered and denoised and divided into Operational Taxonomic Units (OTUs) and (amplicon sequence variants) ASVs. The species classification was based on the sequence composition of the OTUs and ASVs. By comparing the representative sequences of OTUs and ASVs with the Silva database, the confidence score threshold was set to 0.8.

### 2.5 Statistical analysis

SPSS 26 statistical software was used to perform one-way analysis of variance on the differences in various test indicators between groups, expressed as mean ± SD. Differences were considered significant at the *p* < 0.05 level and are represented by different letters in figures. The graphs of physical and chemical indicators, antibiotic resistance gene abundances, and microbial community abundances were completed using Origin 2021. Gene and microbial heatmaps were created using the OmicShare tool located at www.omics.com/tools. Mantel and Spearman correlation analyses were performed using the R language (v4.4.2) and visualised using Gephi software. Partial least squares structural equation modelling (PLS–SEM) was constructed using the R language (v4.4.2) to evaluate the effects of temperature, bacterial communities, and MGEs on ARGs.

## 3 Results

### 3.1 Changes in physical and chemical parameters during the composting process

Temperature is a crucial indicator in the composting process, as it is closely related to microbial activity and organic matter decomposition (Che et al., 2021). All four treatments showed a temperature trend that first increased and then decreased. The days 0–5 period corresponded to the warming phase, during which the compost temperature rapidly increased and peaked, with the highest temperature exceeding 55 °C and lasting for 7 days (Figure 1A). This met the temperature requirements stipulated in the “Technical Specifications for Composting of Livestock and Poultry Manure” (NY/T3442-2019). The M2 group reached its highest temperature of 68 °C on day 3. Both the M1 and M3 treatment groups reached their highest temperatures on day 4, at 66.5 °C and 66.3 °C, respectively. The control group reached a maximum temperature of 63.9 °C on day 6. This indicated that adding CMHRs to the compost shortened the time for reaching the high-temperature stage in the pig manure composting process and increased the maximum temperature. An appropriate pH is crucial for the growth and reproduction of microorganisms and is an important factor determining the maturity of compost (Liu et al., 2023). Compared with the control group, the three treatment groups with the CMHRs exhibited a decreasing-then-increasing pH trend (Figure 1B). When composting was completed, the pH values in the four treatment groups ranged from 7.52 to 8.59. As shown in Figure 1C, the electrical conductivity (EC) values in the control and M1 treatment groups exhibited a decreasing trend, while those in the M2 and M3 treatment groups initially increased and then decreased. At the completion of composting, the EC values in all groups were below 3 mS/cm. The M2 treatment had the lowest EC of 2.53 mS/cm, indicating that M2 treatment was more conducive to reducing the EC of compost.

**Figure 1:**
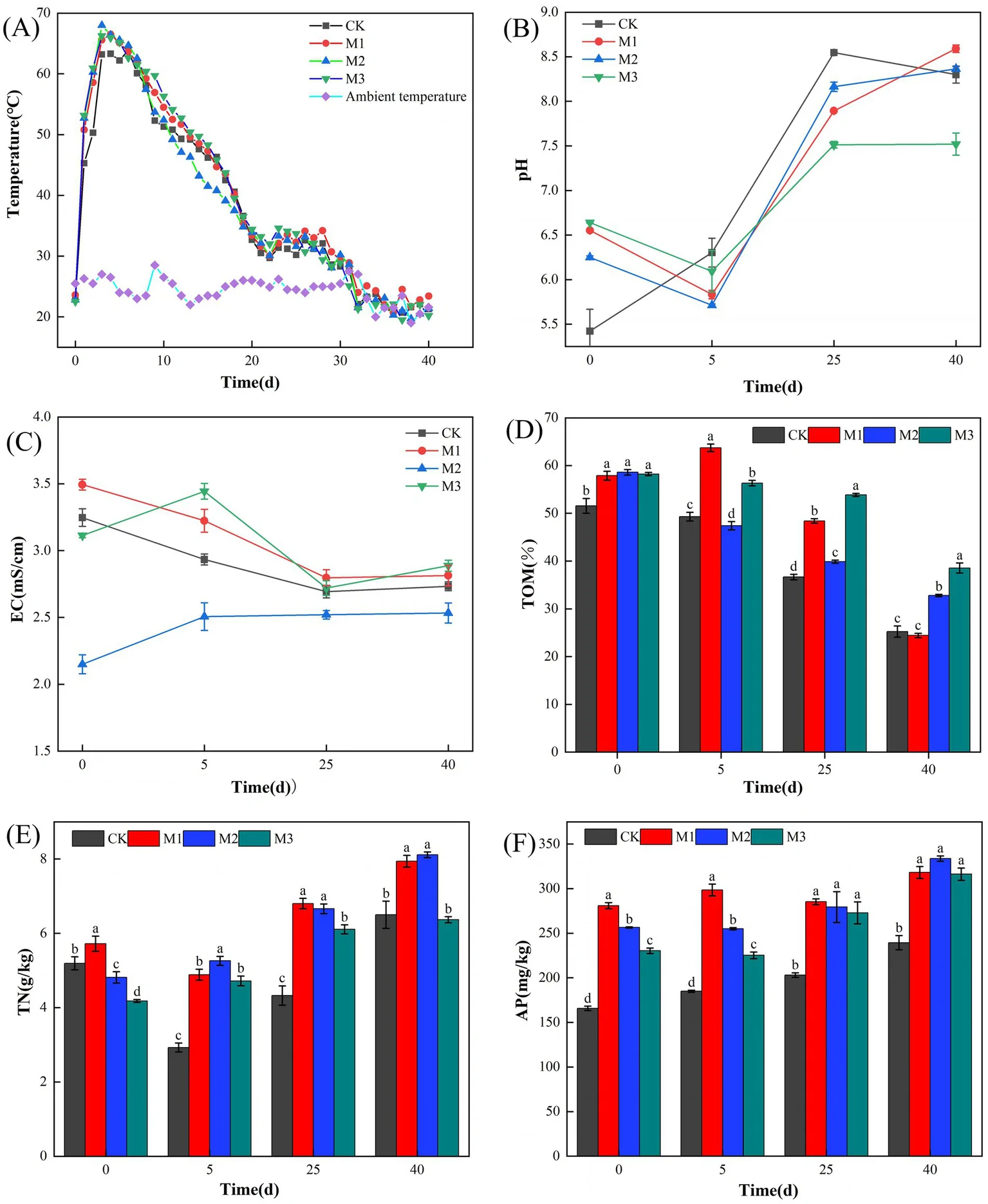
Changes in physical and chemical properties during the composting process: **(A)** temperature, **(B)** pH, **(C)** EC, **(D)** TOM, **(E)** TN, and **(F)** AP. EC, electrical conductivity; TOM, total organic matter; TN, total nitrogen; AP, available phosphorus.

In the composting process, organic matter is mineralised and humified by microorganisms. TOM in all the treatments decreased, mainly from day 5 to day 40 (Figure 1D). After composting, TOMs in the control, M1, M2, and M3 treatment groups were 25.29%, 24.43%, 32.79%, and 38.55%, respectively. As shown in Figures 1E,F, the TN and AP in all four treatment groups ultimately increased. At the end of composting, the TN and AP in the M2 treatment group were significantly higher than those in the control group, at 8.11 g/kg and 333.73 mg/kg, respectively. This suggests that CMHRs added to the pig manure during composting significantly promotes TN and AP accumulation.

### 3.2 Changes in ARGs during the composting process

As shown in Figure 2A, aminoglycoside, β-lactamase, glycopeptide, MLSB, multidrug, phenicol, sulfonamide, and tetracycline ARGs and MGEs were predominant. Throughout the composting process, the total absolute abundance of ARGs in all the treatment groups initially decreased, then increased, and finally decreased. Aerobic composting significantly reduced the total absolute abundance of ARGs. In the initial stage of composting, the total absolute abundances of ARGs in the CK, M1, M2, and M3 groups were 1.34 × 10^10^, 1.41 × 10^10^, 1.18 × 10^10^, and 1.09 × 10^10^ copies/g, respectively, representing reductions of 27.10%, 36.04%, 65.55%, and 40.55%, respectively compared to the end of composting. Compared with the control group, the groups with CMHRs exhibited a significantly reduced absolute abundance of ARGs. The removal rate of aminoglycoside resistance genes was highest in the M2 treatment group, at 78.09%, while in the CK, M1, and M3 groups, the removal rate was 20.32%, 45.55%, and 61.94%, respectively. The removal rates of β-lactam resistance genes in the CK, M1, M2, and M3 groups were 30.60%, 54.93%, 81.11%, and 24.09%, respectively. In the CK, M1, M2, and M3 groups, the removal rates of multidrug resistance genes were 39.93%, 46.38%, 74.09%, and 30.67%, respectively. In the CK, M1, M2, and M3 groups, the removal rates of sulfonamide resistance genes were 17.77%, 31.49%, 54.27%, and 31.97%, and those of tetracycline resistance genes were 43.52%, 22.92%, 61.24%, and 41.36%, respectively. Furthermore, among all the resistance gene categories, the removal rate of glycopeptide resistance genes was the most significant, reaching over 90% in all but the M2 treatment group. The removal rates of MGEs in the CK, M1, M2, and M3 groups were 19.25%, 56.20%, 65.32%, and 14.03%, respectively. Interestingly, at the end of composting, the absolute abundance of diaminopyrimidine resistance genes increased by 176.07%, 224.21%, 50.04 and 179.64%, respectively, compared with that of the control group.

**Figure 2:**
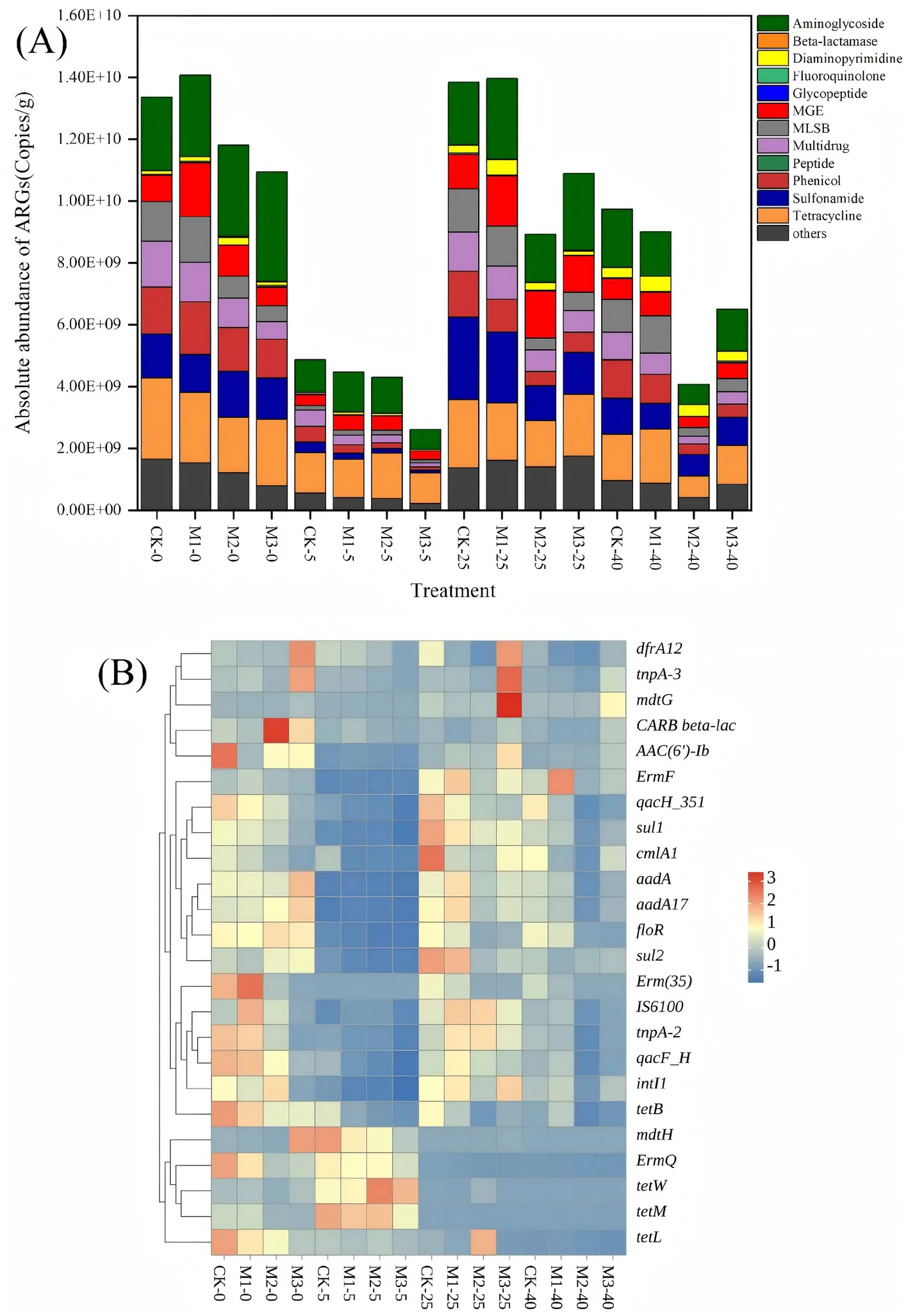
Changes in the antibiotic resistance genes, with the **(A)** absolute abundance and **(B)** absolute abundance changes as a heatmap of typical ARGs during the composting process: *aadA*, *aadA17*, *AAC(6′)-Ib*, *CARB*, *dfrA12*, *mdtH*, *IS6100*, *intI1*, *tnpA-3*, *tnpA-2*, *ErmQ*, *ErmF*, *Erm(35)*, *mdtG*, *qacF_H*, *qacH_351*, *floR*, *cmlA1*, *sul1*, *sul2*, *tetB*, *tetL*, *tetW*, *tetM*). ARGs, antibiotic resistance genes.

Changes in the abundance of 24 typical antibiotic resistance genes in the compost were further analysed (Figure 2B). The abundance of most of the resistance genes significantly decreased after composting. Overall, compared with the control, the CMHRs treatment effectively reduced the ARGs. M2 treatment produced a lower abundance of ARGs than the other treatments. For example, the removal rates of aminoglycoside resistance genes *aadAa* and *adA17* were 32.39 and 29.58% (M1 group), 77.09 and 78.11% (M2 group), 67.47 and 62.15% (M3 group), and 17.99 and 8.03% (CK group), respectively. The removal rates of *dfrA12* (diaminopyrimidine resistance gene) were 28.16%, 80.79%, 89.49%, and 76.73% in the CK, M1, M2, and M3 groups, respectively. The removal rates of *mdtH* (fluoroquinolone resistance gene) were 96.99%, 92.74%, 70.61%, and 99.81% after CK, M1, M2, and M3 treatment, respectively. In contrast, after composting, a multidrug resistance gene *mdtG* was amplified with CK, M1, M2, and M3 treatments, with an increase of 94.35%, 92.51%, 71.64%, and 302.22%, respectively. The removal rates of the phenicol resistance genes *floR* and *cmlA1* were 12.01% and −17.43% (CK group), 20.69 and 47.47% (M1 group), 72.83 and 74.47% (M2 group), 73.92% and −190.94%, (M3 group), respectively. Similarly, the removal rates of the sulfonamide resistance genes *sul1* and *sul2* were 21.59 and 14.22% (CK group), 28.42 and 34.80% (M1 group), 63.15 and 49.40% (M2 group), and −10.97 and 45.80% (M3 group), respectively. The removal rates of tetracycline resistance genes, including *tetB*, *tetL*, *tetW*, and *tetM*, were nearly all above 90% in all groups. The removal rates of *IS6100*, *intI1*, *tnpA-3*, and *tnpA-2* (MGEs) were higher in the M2 treatment group than in the control group, reaching 67.79%, 73.77%, 77.70%, and 67.31%, respectively.

### 3.3 Changes in bacterial communities during the composting process

Changes in the bacterial community phylum levels during the composting process are shown in Figure 3A. In this experiment, the Firmicutes, Proteobacteria, Actinobacteria, and Bacteroidetes were the four major phyla, accounting for 76.08 to 99.86% of the total bacterial abundance in each group. After the composting process ended, the relative abundance of the Firmicutes decreased. After CK, M1, M2, and M3 treatments, the relative abundance decreased by 44.42%, 75.03%, 72.83%, and 78.72%, respectively. During composting, the relative abundance of the Proteobacteria phylum increased. After composting, compared with the control, the M2 and M3 treatment groups exhibited a significant increase in relative abundance. The relative abundance of the Actinobacteria phylum had an overall trend exhibiting an increase and then a decrease. The highest abundance in the M3 treatment group was on day 25 at 31.58%. After composting, the relative abundances in the CK, M1, M2, and M3 treatment groups were 9.66%, 12.83%, 21.85%, and 20.58%, respectively. The relative abundance of the Bacteroidetes was the lowest during the high temperature period and increased during the cooling period.

**Figure 3:**
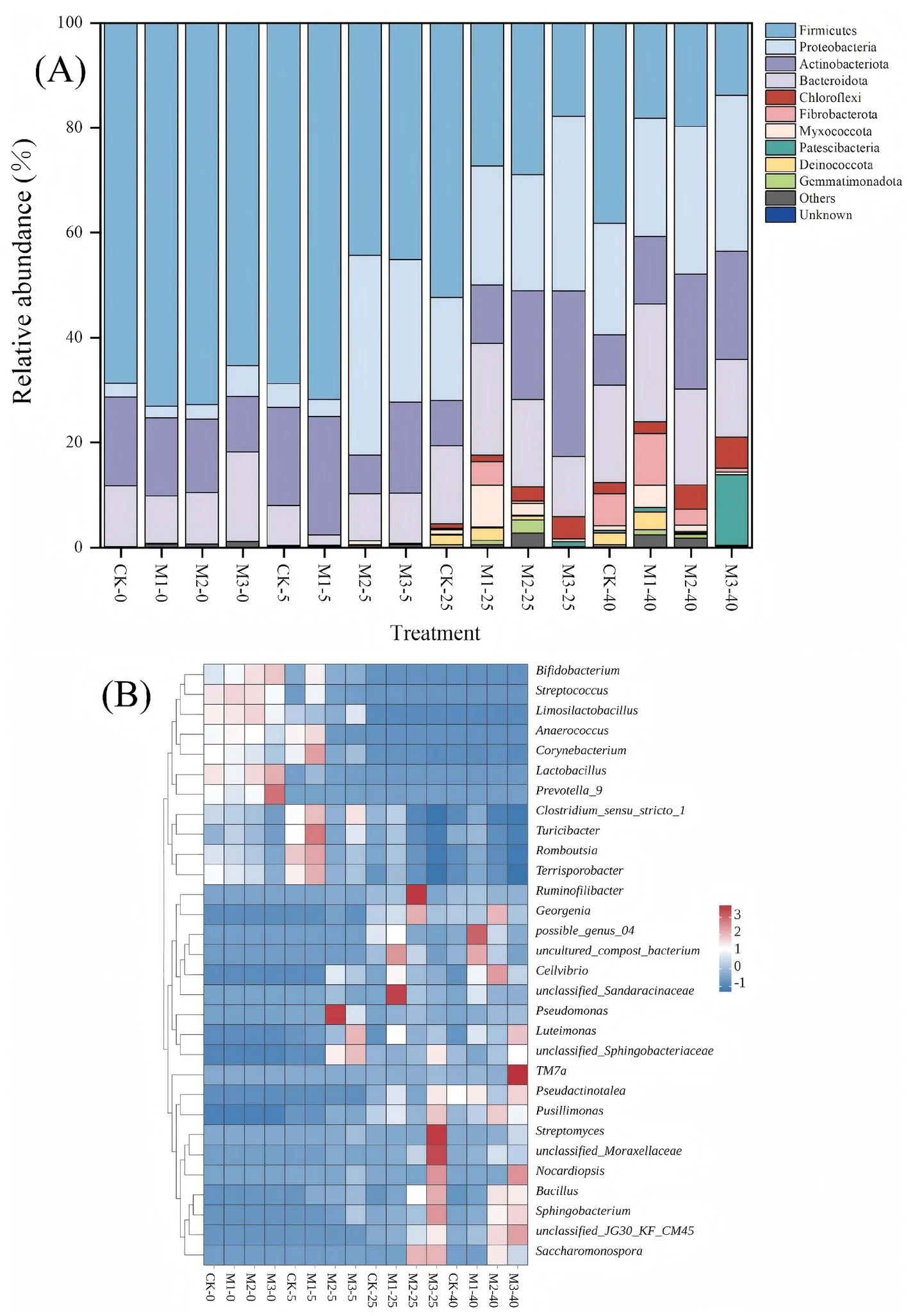
Changes in the bacterial community abundance at the phylum **(A)** and genus **(B)** levels during the composting process.

To further investigate the changes in the bacterial communities, a heatmap was generated of the top 30 microbial clusters at the genus level with their relative abundances throughout the composting process (Figure 3B). In the raw materials, the abundances of *Clostridium sensu stricto* 1 (Firmicutes), *Corynebacterium* (Actinobacteriota), *Georgenia* (Actinobacteriota), *Lactobacillus* (Firmicutes), *Streptococcus* (Firmicutes), *Terrisporobacter* (Firmicutes), and *Turicibacter* (Firmicutes) were relatively high, but significantly decreased after composting. Among them, the relative abundances of *Clostridium sensu stricto* 1 decreased by 38.66%, 96.47%, 71.52%, and 97.78% and those of *Corynebacterium* decreased by 72.54%, 98.91%, 71.39%, and 93.77% with CK, M1, M2, and M3 treatment, respectively. The relative abundance of *Turicibacteria* decreased to 9.85%, 29.11%, 66.99%, and 80.81% following CK, M1, M2, and M3 treatment, respectively. After composting, the relative abundance of Streptococcus decreased to almost zero in all treatments. However, the relative abundance of other bacterial genera, such as *Bacillus* (Firmicutes) and *Pseudomonas* (Proteobacteria), increased after composting. These results indicate that CMHRs alter the microbial population structure in pig manure compost, and different concentrations of CMHRs produce different effects on the microbial population structure. It was speculated that changes in the ARG abundance may be related to the different patterns of these bacterial genera.

### 3.4 Effects of physicochemical indicators on ARGs and bacterial communities during composting

Results of a correlation analysis between the ARGs and MGEs and the physicochemical indicators are presented in Figure 4. Correlations were observed among different parameters, with temperature positively correlated with TOM and EC and negatively correlated with TN, AP, and pH. TOM was significantly negatively correlated with TN (*p* < 0.05) and pH (*p* < 0.01). TN was significantly positively correlated with AP (*p* < 0.001) and pH (*p* < 0.05), and ARGs were significantly correlated with temperature (*p* < 0.01). The bacterial community was sensitive to changes in the physicochemical properties, with significant correlations with TOM, TN, pH, and EC (*p* < 0.05). However, the correlation between the MGEs and physicochemical indicators was not significant.

**Figure 4:**
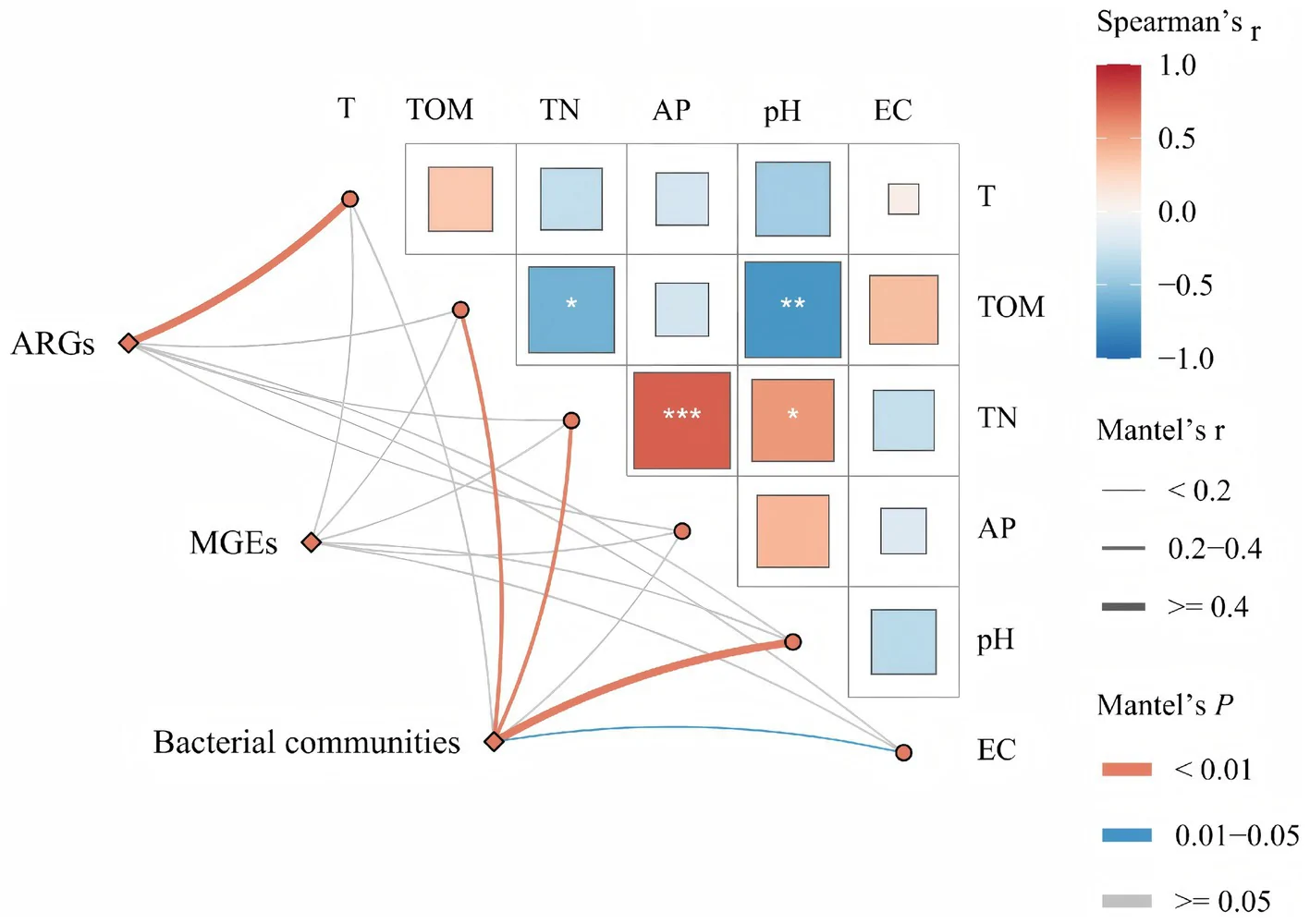
Mantel test results of correlations among ARGs, MGEs, microbial communities, and physicochemical indicators during the composting process. ARGs, antibiotic resistance genes; MGEs, mobile genetic elements.

### 3.5 Co-occurrence of the ARGs, MGEs, and bacterial communities

To investigate the relationships among the microorganisms, ARGs, and MGEs at the genus level, a network analysis was conducted based on Spearman correlation coefficients (|*r*| > 0.8, *p* < 0.01). As shown in Figure 5, the network consisted of 47 nodes, including 15 ARGs, 3 MGEs, and 185 edges. Correlations among 28 bacterial genera, 16 ARGs, and 3 MGEs were obtained. Research has shown that host bacteria significantly positively correlated with ARGs include mainly Firmicutes and Actinobacteria. Furthermore, a single ARG may have multiple potential hosts, and a single host bacteria may carry multiple ARGs. For example, both *tetM* and *ErmQ* were significantly positively correlated with *Clostridium sensu stricto* 1, *Corynebacterium*, *Terrisporobacter*, *Anaerococcus*, and *Romboutsia*, and *mdtH* and *Corynebacterium* were also significantly positively correlated, indicating that these bacterial genera may potentially host these genes. In addition, *intI1* was significantly positively correlated with sul1 and *qacF-H*, while *tnpA-2* was significantly positively correlated with *sul1*, *Erm (35)*, and *qacH351*.

**Figure 5:**
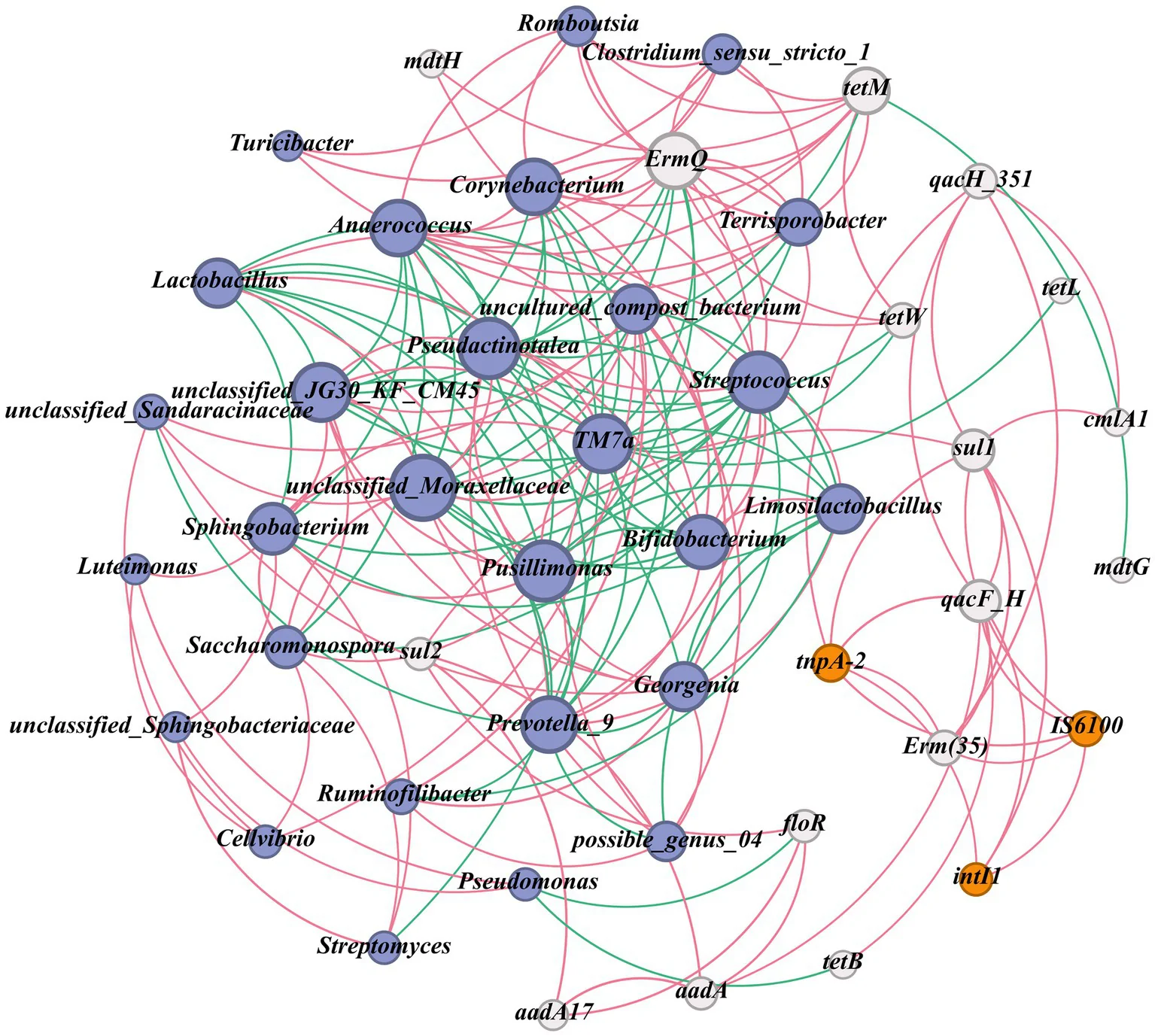
Network analysis of the ARGs, MGEs, and bacterial communities (top 30 genera). The connection represents a significant correlation based on a Spearman correlation analysis (|*r*| > 0.8, *p* < 0.01), in which the node size is proportional to the node degree. Red and green lines represent positive and negative correlations, respectively. The thickness of the line is directly proportional to the absolute value of the correlation coefficient. ARGs, antibiotic resistance genes; MGEs, mobile genetic elements.

### 3.6 Correlations among temperature, ARGs, MGEs, and the microbial community during the composting process

The relationships among temperature, microbial community, MGEs, and ARGs were further explored using partial least squares structural equation modelling (PLS-SEM). As shown in Figure 6, both temperature and MGEs had a direct and significant effect on the ARGs, with the MGEs having the most significant effect (MGEs, *p* < 0.001; temperature, *p* < 0.01). This indicated that changes in ARGs during composting may be closely related to changes in temperature and MGEs. In addition, temperature was negatively correlated with bacterial communities, which directly affect MGEs and ultimately impact ARGs during composting.

**Figure 6:**
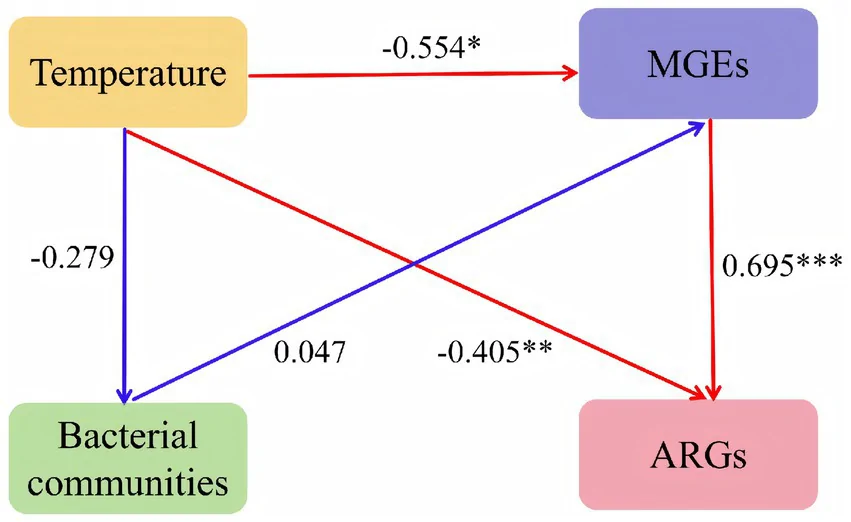
The structural equation model (SEM) reflects the direct and indirect effects of the composting temperature, microbial community, and MGEs on ARGs. The red and black arrows represent positive and negative correlations, respectively. Significance level: ^*^p < 0.05, ^**^p < 0.01, ^***^p < 0.001. ARGs, antibiotic resistance genes; MGEs, mobile genetic elements.

## 4 Discussion

The results of this study demonstrated the effects of adding CMHRs on changes of ARGs and MGEs during the aerobic composting of pig manure their relationships with physicochemical parameters and microbial communities. The addition of 30% CMHRs (M2 treatment) effectively reduced ARGs and MGEs in pig manure compost. Temperature, MGEs, organic carbon, TN, and microbial communities were associated to varying degrees with changes in ARGs.

Temperature is an important parameter affecting compost maturity and microbial activity. In particular, during the high-temperature stage, pathogenic microorganisms can be inactivated and insect eggs and weed seeds can be eliminated from the compost (Zhang et al., 2019). In this experiment, the temperatures of all treatments met the requirements of the Chinese “Technical Specification for Livestock and Poultry Manure Composting” (NY/T3442-2019). Furthermore, incorporating CMHRs into the compost significantly shortened the time required to reach the high-temperature stage and increased the maximum temperature compared with the control group. High temperatures may disrupt antibiotic structures, thereby contributing to the reduction of ARGs (Liu et al., 2023).

The four treatments showed similar trends in pH. The pH was initially low, subsequently increased, and tended to stabilise during the later stages of composting. This may be related to phenolic compound synthesis, NO2^−^ accumulation, and ammonia release during composting (Wu et al., 2024). EC is a crucial parameter for assessing compost quality and maturity. High EC values in compost can exert toxic effects (Liu et al., 2018). The EC values in the control and M1 treatments exhibited a decreasing trend during composting, whereas those in the M2 and M3 treatments initially increased and then decreased. This phenomenon may be associated with processes occurring during composting, such as ammonia gas volatilisation, salt ion precipitation, and organic acid degradation (Mao et al., 2019). The EC values in all treatments remained below 3 mS/cm, thereby meeting the standards for agricultural application.

The mineralisation of organic matter during composting is closely associated with microbial activity and changes in ARGs. TOM was relatively high at the beginning of composting in all treatment groups but decreased continuously as composting progressed. This may reflect the continuous consumption and decomposition of organic matter by microorganisms and is consistent with previous findings (Qin et al., 2024). Regarding nutrient content, TN and AP ultimately increased in all four treatments eventually showed an upward trend. Moreover, CMHRs-amended treatments showed higher final TN and AP concentrations than the control, suggesting improved apparent nutrient retention.

The addition of traditional CMHRs during composting significantly affected ARG abundance. As shown in Figure 2A, ARGs conferring resistance to aminoglycoside, β-lactams, glycopeptide, MLSB, multiple drugs, phenicol, sulphonamide, and tetracyclines, as well as MGEs, predominated. During composting, their levels initially increased and then decreased. ARG removal may occur primarily during the high-temperature stage, likely due to the inactivation of thermophilic antibiotic-resistant host microorganisms (Zhang W. et al., 2023). The subsequent increase during the maturation stage may results from the survival and proliferation of dormant antibiotic-resistant bacteria and increased horizontal gene transfer (Zhu et al., 2022). Studies have shown that different additives may have varying effects on the types and abundance of ARGs removed during composting. For example, at the end of composting, a low carbon/nitrogen ratio and the additiona of high-quality lignin significantly reduced the relative abundance of *tetW*, *ermB*, *ermC*, *sul1*, and *sul2* (Qiu et al., 2021). The addition of nano-zero-valent iron during the maturation stage of composting resulted in removal rates exceeding 97% for the total abundance of intracellular and extracellular ARGs and MGEs (Zhou et al., 2023).

Similarly, the present results showed that the addition of CMHRs significantly reduced the absolute abundance of ARGs and MGEs. The M2 treatment achieved the highest removal rates of aminoglycoside, β-lactam, multidrug resistance, sulfonamide, and tetracycline resistance genes and MGEs at 78.09%, 81.11%, 74.09%, 54.27%, 61.24%, and 65.32%, respectively. The addition of CMHRs altered the compost microbial community structure and reduced the abundance of most ARGs, consistent with the findings of Zhang et al. (2017). Bioactive antibacterial compounds in CMHRs may alter microbial community structure by modifying their metabolic environment, thereby potentially inhibiting the proliferation and spread of ARGs.

The abundance of certain resistance genes increase after composting is completed. A previous study showed that biochar added to a sludge composting significantly increased the abundance of the multidrug resistance gene adeF at the mature stage, and that adding 400 °C pyrolysis biochar to the compost inhibited the expression of *adeF* (Cheng et al., 2025b). In this study, *mdtG* levels increased after composting by 94.35%, 92.51%, 71.64%, and 302.22% with the CK, M1, M2, and M3 treatments, respectively. *mdtG* is a bacterial multidrug resistance efflux pump gene. Previous studies have confirmed that phenolic acid secondary metabolites can induce the expression of multiple efflux pumps in bacteria (Blanco et al., 2016; Ravirala et al., 2007). In this experiment, the increased abundance of *mdtG* may be associated with the accumulation of phenolic acids generated during composting. However, the high content of secondary metabolites such as alkaloids and polyphenols released from high added CMHRs may further exacerbate the increase in the abundance of *mdtG*.

Microorganisms are affected by environmental factors and have an important impact on composting efficiency and abundance of ARGs and MGEs. In this study, Firmicutes, Proteobacteria, Actinobacteria, and Bacteroidetes were the four main phyla present in the compost, consistent with the findings of Yan et al. (2025). Firmicutes and Actinobacteria are natural producers of antibiotics and are responsible for spreading and carrying ARGs (Huerta et al., 2013). Therefore, changes in these bacterial phyla may alter ARGs. Among the physicochemical indicators that affect ARGs and microbial communities, temperature is significantly correlated with ARGs, while TOM, TN, pH, and EC are significantly correlated with microbial communities. High temperature can reduce the abundance of ARGs by destroying their host bacteria, degrading MGEs, and reshaping microbial communities (Sun et al., 2016; Wang et al., 2022). This is consistent with the finding that ARG abundance significantly decreased during the high-temperature stage in this study.

Carbon and nitrogen are essential nutrients for microbial proliferation. Their concentrations can influence the enrichment of different microbial communities according to their nutritional requirements, thereby affecting the composition of potential host microorganisms carrying ARGs and MGEs (Wang B. et al., 2024). The network analysis performed in this study revealed that *tetM* and *ErmQ* were significantly positively correlated with *Clostridium sensu stricto* 1, *Corynebacterium*, *Terrisporobacter*, *Anaerobacter*, and *Romboutsia*, whereas *mdtH* was significantly positively correlated with *Bacillus*, suggesting that these bacterial genera may be potential hosts of the corresponding genes. Furthermore, *intI1* was significantly positively correlated with *sul1* and *qacF-H*, while *tnpA-2* was significantly positively correlated with *sul1*, *Erm(35)*, and *qacH3511*. These associations suggest that MGEs may contribute to the dissemination of ARGs.

The PLS-SEM results further showed that temperature and MGEs had significant direct effects on ARGs. This finding was consistent with previous reports suggesting that changes in MGEs and environmental factors are associated with changes in ARGs (Liao et al., 2017; Qiu et al., 2021). In addition, physicochemical parameters had indirect effects on ARGs, potentially because changes in these parameters, particularly the carbon-to-nitrogen ratio, can affect the growth of microorganisms associated with ARGs and MGEs during composting (Wei et al., 2020). In summary, the results of this experiment indicated that temperature and physicochemical properties were directly or indirectly associated with changes in ARGs during composting. Therefore, during aerobic composting of livestock manure with CMHRs, regulating environmental factors and optimising the characteristics of the added substrate may facilitate ARG removal and reduce the environmental risks associated with the agricultural application of organic fertilisers.

This study highlights the potential role of CMHRs in reducing the abundance of ARGs and MGEs during the aerobic composting of pig manure, but it has some limitations. First, the experiment was conducted using small-scale composting reactor composting reactors. Environmental conditions may fluctuate more substantially at large-scale composting sites; therefore, the effects observed in this study require further validation under practical production conditions. Second, the CMHRs used in this study were derived from *Cistanche deserticola* and Fructus Aurantii immaturus, and the specific components responsible for the observed effects remain unknown. Moreover, the bioactive components of CMHRs from different sources may vary considerably, potentially resulting in different effects on ARGs during pig manure composting. Further studies are needed to determine how these components interact with microbial communities and environmental factors during composting.

Future studies should screen CMHRs raw materials containing different bioactive components and investigate how the active substances, such as polyphenols and alkaloids, affect potential ARG hosts and horizontal gene transfer. Integrating metabolomic analyses may further clarify the underlying molecular mechanism. Such studies could facilitate the resource utilisation of CMHRs and help improve the safety of compost products.

## 5 Conclusion

This study investigated changes in physicochemical properties, ARGs, MGEs, and microbial communities during pig manure composting with different proportions of CMRHs derived from Cistanches Herba and Aurantii Fructus Immaturus. The addition of CMHRs significantly reduced the abundance of ARGs and MGEs, particularly when CMRHs constituted 30% CMHRs of the initial compost mixture by mass, with total ARG and MGE removal rates exceeding 65%. The addition of CMHRs also significantly altered the microbial communities, including Firmicutes, Bacteroidetes, Actinobacteria, and Proteobacteria, some members of which may serve as potential hosts for ARGs and MGEs. PLS-SEM indicated that temperature and MGEs had significant direct effects on ARGs, whereas TOM and TN had indirect effects mediated by microbial communities. These findings provide a basis for the use of CMHRs to reduce the spread of ARGs during pig manure composting.

## Data Availability

The high-throughput sequencing results from this experiment have been submitted to CNCB under GSA number CRA050065.

## References

[ref1] Blanco P. Hernando-Amado S. Reales-Calderon J. Corona F. Lira F. Alcalde-Rico M. . (2016). Bacterial multidrug efflux pumps: much more than antibiotic resistance determinants. Microorganisms 4:14. doi: 10.3390/microorganisms4010014, 27681908 PMC5029519

[ref2] Cai R. Xu Q. Luo X. Xiong G. Wang C. Yu W. . (2025). Influence of turning frequency on removal of pathogens and antibiotic resistance genes from full-size sheep manure composting on the Qinghai-Tibet plateau. Biochem. Eng. J. 222:109845. doi: 10.1016/j.bej.2025.109845

[ref4] Cao Y. Zhou C. Liu G. (2025). Study on the effect of straw addition ratio on antibiotic resistance genes in rapid production of cattle manure organic fertilizer. Environ. Technol. 47, 698–711. doi: 10.1080/09593330.2025.2597557 41348588

[ref5] Che J. Bai Y. Li X. Ye J. Liao H. Cui P. . (2021). Linking microbial community structure with molecular composition of dissolved organic matter during an industrial-scale composting. J. Hazard. Mater. 405:124281. doi: 10.1016/j.jhazmat.2020.124281, 33097342

[ref6] Chen M. Wang C. Wang B. Bai X. Huang Y. (2019). Enzymatic mechanism of organic nitrogen conversion and ammonia formation during vegetable waste composting using two amendments. Waste Manag. 95, 306–315. doi: 10.1016/j.wasman.2019.06.027, 31351616

[ref7] Chen Z. Bao H. Wen Q. Wu Y. Fu Q. (2022). Effects of H3PO4 modified biochar on heavy metal mobility and resistance genes removal during swine manure composting. Bioresour. Technol. 346:126632. doi: 10.1016/j.biortech.2021.126632, 34971775

[ref8] Cheng D. Jiang Y. Chen J. Lin H. Yu Z. (2025a). Enhancing compost safety: simultaneous reduction of antibiotic resistance genes and bioavailable heavy metals by microbial additives. Bioresour. Technol. 443:133826. doi: 10.1016/j.biortech.2025.133826, 41423071

[ref9] Cheng D. Xiong J. Chen J. Chang H. Wong J. W. C. (2025b). Effect of biochar addition on antibiotic and heavy metal resistance genes during sewage sludge composting. J. Environ. Chem. Eng. 13:115732. doi: 10.1016/j.jece.2025.115732

[ref10] Guo B. Wu J. Chen J. Zhang H. Li J. (2022). Effects of Chinese medicine herbal residues on antibiotic resistance genes and the bacterial community in chicken manure composting. J. Antibiot. 75, 164–171. doi: 10.1038/s41429-022-00505-x, 35058576

[ref11] Hou X. Deng Y. Dai M. Jiang X. Li S. Fu H. . (2022). Migration and transformation of heavy metals in Chinese medicine residues during the process of traditional pyrolysis and solar pyrolysis. Chemosphere 293:133658. doi: 10.1016/j.chemosphere.2022.133658, 35051513

[ref3] Huang C. Tang Z. Xi B. Tan W. Guo W. Wu W. . (2021). Environmental effects and risk control of antibiotic resistance genes in the organic solid waste aerobic composting system: a review. Front. Environ. Sci. Eng. 15:127. doi: 10.1007/s11783-021-1415-5

[ref12] Huerta B. Marti E. Gros M. López P. Pompêo M. Armengol J. . (2013). Exploring the links between antibiotic occurrence, antibiotic resistance, and bacterial communities in water supply reservoirs. Sci. Total Environ. 456–457, 161–170. doi: 10.1016/j.scitotenv.2013.03.071, 23591067

[ref13] Li J. Lu H. Yang H. Wen X. Huang Y. Li Q. (2023). Performances of antibiotic resistance genes profile upon the action of biochar-activated peroxydisulfate in composting. J. Environ. Manag. 334:117509. doi: 10.1016/j.jenvman.2023.117509, 36801799

[ref14] Li P. Zhao Z. Zhang M. Su H. Zhao T. Feng W. . (2024). Exploring the potential of biochar derived from chinese herbal medicine residue for efficient removal of norfloxacin. Molecules 29:2063. doi: 10.3390/molecules29092063, 38731553 PMC11085230

[ref15] Liao H. Lu X. Rensing C. Friman V. P. Geisen S. Chen Z. . (2017). Hyperthermophilic composting accelerates the removal of antibiotic resistance genes and mobile genetic elements in sewage sludge. Environ. Sci. Technol. 52, 266–276. doi: 10.1021/acs.est.7b04483, 29199822

[ref16] Liu H. Awasthi M. K. Zhang Z. Syed A. Bahkali A. H. Sindhu R. . (2023). Microbial dynamics and nitrogen retention during sheep manure composting employing peach shell biochar. Bioresour. Technol. 386:129555. doi: 10.1016/j.biortech.2023.129555, 37499921

[ref17] Liu H. Zhou Y. Qin S. Awasth S. K. Liu T. Liu H. . (2021). Distribution of heavy metal resistant bacterial community succession in cow manure biochar amended sheep manure compost. Bioresour. Technol. 335:125282. doi: 10.1016/j.biortech.2021.125282, 34020875

[ref18] Liu L. Wang S. Guo X. Zhao T. Zhang B. (2018). Succession and diversity of microorganisms and their association with physicochemical properties during green waste thermophilic composting. Waste Manag. 73, 101–112. doi: 10.1016/j.wasman.2017.12.026, 29279244

[ref19] Liu R. Yang Z. Wang G. Junren X. Li T. Pu Y. . (2021). Simultaneous removal of ammonium and phosphate in aqueous solution using Chinese herbal medicine residues: mechanism and practical performance. J. Clean. Prod. 313:127945. doi: 10.1016/J.JCLEPRO.2021.127945

[ref20] Lu Q. Li C. (2020). Comprehensive utilization of Chinese medicine residues for industry and environment protection: turning waste into treasure. J. Clean. Prod. 279:123856. doi: 10.1016/j.jclepro.2020.123856

[ref21] Ma R. Wang J. Liu Y. Wang G. Yang Y. Liu Y. . (2023). Dynamics of antibiotic resistance genes and bacterial community during pig manure, kitchen waste, and sewage sludge composting. J. Environ. Manag. 345:118651. doi: 10.1016/j.jenvman.2023.118651, 37499413

[ref22] Mao H. Zhang H. Fu Q. Zhong M. Li R. Zhai B. . (2019). Effects of four additives in pig manure composting on greenhouse gas emission reduction and bacterial community change. Bioresour. Technol. 292:121896. doi: 10.1016/j.biortech.2019.121896, 31404757

[ref23] Qi J. Yang H. Wang X. Zhu H. Wang Z. Zhao C. . (2023). State-of-the-art on animal manure pollution control and resource utilization. J. Environ. Chem. Eng. 11:110462. doi: 10.1016/j.jece.2023.110462

[ref24] Qin J. Wang M. Zhou J. Tong Z. Li L. Liu F. (2024). Effects of adding corn straw and apple tree branches on antibiotic resistance genes removal during sheep manure composting. J. Environ. Manag. 370:122910. doi: 10.1016/j.jenvman.2024.122910, 39405860

[ref25] Qiu X. Zhou G. Chen L. Wang H. (2021). Additive quality influences the reservoir of antibiotic resistance genes during chicken manure composting. Ecotoxicol. Environ. Saf. 220:112413. doi: 10.1016/j.ecoenv.2021.112413, 34139628

[ref26] Qiu X. Zhou G. Wang H. (2022). Nanoscale zero-valent iron inhibits the horizontal gene transfer of antibiotic resistance genes in chicken manure compost. J. Hazard. Mater. 422:126883. doi: 10.1016/j.jhazmat.2021.126883, 34416685

[ref27] Qiu Z. Fu K. Yu D. Luo J. Shang J. Luo S. (2022). Radix Astragali residue-derived porous amino-laced double-network hydrogel for efficient Pb (II) removal: performance and modeling. J. Hazard. Mater. 438:129418. doi: 10.1016/j.jhazmat.2022.129418, 35780735

[ref28] Ravirala R. S. Barabote R. D. Wheeler D. M. Reverchon S. Tatum O. Malouf J. . (2007). Efflux pump gene expression in *Erwinia chrysanthemi* is induced by exposure to phenolic acids. Mol. Plant-Microbe Interact. 20, 313–320. doi: 10.1094/MPMI-20-3-0313, 17378434

[ref29] Ren X. Wang Q. Awasthi M. K. Zhao J. Wang J. Liu T. . (2019). Improvement of cleaner composting production by adding diatomite: from the nitrogen conservation and greenhouse gas emission. Bioresour. Technol. 286:121377. doi: 10.1016/j.biortech.2019.121377, 31039466

[ref30] Sun W. Qian X. Gu J. Wang X. Duan M. (2016). Mechanism and effect of temperature on variations in antibiotic resistance genes during anaerobic digestion of dairy manure. Sci. Rep. 6:30237. doi: 10.1038/srep30237, 27444518 PMC4957233

[ref31] Wang B. Chen W. Sa C. Gao X. Chang S. Wei Y. . (2024). Dynamics of antibiotic resistance genes and the association with bacterial community during pig manure composting with chitin and glucosamine addition. Front. Microbiol. 15:1384577. doi: 10.3389/fmicb.2024.1384577, 38841060 PMC11150687

[ref32] Wang G. Gao X. Cai Y. Li G. Ma R. Yuan J. (2024a). Dynamics of antibiotic resistance genes during manure composting: reduction in herbivores manure and accumulation in carnivores. Environ. Int. 190:108900. doi: 10.1016/j.envint.2024.108900, 39053194

[ref33] Wang G. Kong Y. Yang Y. Ma R. Li L. Li G. . (2022). Composting temperature directly affects the removal of antibiotic resistance genes and mobile genetic elements in livestock manure. Environ. Pollut. 303:119174. doi: 10.1016/j.envpol.2022.119174, 35306090

[ref34] Wang G. Liu R. Yang Z. Liu W. Xu X. Zhang S. . (2024b). Enhanced simultaneous removal of phosphate and ammonium from swine wastewater using magnetic magnesium–loaded Chinese herbal medicine residues: performance, mechanism, and resource utilization. Bioresour. Technol. 393:130103. doi: 10.1016/j.biortech.2023.130103, 38008222

[ref35] Wang H. Lou X. Hu Q. Sun T. (2021). Adsorption of antibiotics from water by using Chinese herbal medicine residues derived biochar: preparation and properties studies. J. Mol. Liq. 325:114967. doi: 10.1016/j.molliq.2020.114967

[ref36] Wang J. Ben W. Zhang Y. Yang M. Qiang Z. (2015). Effects of thermophilic composting on oxytetracycline, sulfamethazine, and their corresponding resistance genes in swine manure. Environ. Sci.: Processes Impacts 17, 1654–1660. doi: 10.1039/c5em00132c, 26216606

[ref37] Wang Q. Wang Z. Awasthi M. K. Jiang Y. Li R. Ren X. . (2016). Evaluation of medical stone amendment for the reduction of nitrogen loss and bioavailability of heavy metals during pig manure composting. Bioresour. Technol. 220, 297–304. doi: 10.1016/j.biortech.2016.08.081, 27589824

[ref38] Wei H. Ma J. Su Y. Xie B. (2020). Effect of nutritional energy regulation on the fate of antibiotic resistance genes during composting of sewage sludge. Bioresour. Technol. 297:122513. doi: 10.1016/j.biortech.2019.122513, 31821955

[ref39] Wu H. Sun X. Liang B. Chen J. Zhou X. (2020). Analysis of livestock and poultry manure pollution in China and its treatment and resource utilization. J. Agro Environ. Sci. 39, 1168–1176.

[ref40] Wu N. Xie S. Zeng M. Xu X. Li Y. Liu X. . (2020). Impacts of pile temperature on antibiotic resistance, metal resistance and microbial community during swine manure composting. Sci. Total Environ. 744:140920. doi: 10.1016/j.scitotenv.2020.140920, 32711322

[ref41] Wu Z. Zhang L. Lin H. Zhou S. (2024). Enhanced removal of antibiotic resistance genes during chicken manure composting after combined inoculation of *Bacillus subtilis* with biochar. J. Environ. Sci. 135, 274–284. doi: 10.1016/j.jes.2022.12.002, 37778803

[ref42] Xu M. Wang F. Stedtfeld R. D. Fu Y. Xiang L. Sheng H. . (2024). Transfer of antibiotic resistance genes from soil to rice in paddy field. Environ. Int. 191:108956. doi: 10.1016/j.envint.2024.108956, 39190978

[ref43] Yan G. Hao Q. Gao Z. Sun Y. Xue X. Qian X. . (2025). Enhanced removal of high-risk antibiotic resistance genes in co-composting by applying compound functional microbial inoculant. Chem. Eng. J. 515:163627. doi: 10.1016/j.cej.2025.163627

[ref44] Yu P. Dong P. Zou Y. Wang H. (2023). Effect of pH on the mitigation of extracellular/intracellular antibiotic resistance genes and antibiotic resistance pathogenic bacteria during anaerobic fermentation of swine manure. Bioresour. Technol. 373:128706. doi: 10.1016/j.biortech.2023.128706, 36746211

[ref45] Zhang K. Gu J. Wang X. Zhang X. Hu T. Zhao W. (2019). Analysis for microbial denitrification and antibiotic resistance during anaerobic digestion of cattle manure containing antibiotic. Bioresour. Technol. 291:121803. doi: 10.1016/j.biortech.2019.121803, 31326686

[ref46] Zhang L. Gu J. Wang X. Sun W. Yin Y. Sun Y. . (2017). Behavior of antibiotic resistance genes during co-composting of swine manure with Chinese medicinal herbal residues. Bioresour. Technol. 244, 252–260. doi: 10.1016/j.biortech.2017.07.035, 28780258

[ref48] Zhang W. Yu C. Yin S. Chang X. Chen K. Xing Y. . (2023). Transmission and retention of antibiotic resistance genes (ARGs) in chicken and sheep manure composting. Bioresour. Technol. 382:129190. doi: 10.1016/j.biortech.2023.129190, 37196739

[ref49] Zhang Y. Wang N. Wan J. Jousset A. Jiang G. Wang X. . (2024). Exploring the antibiotic resistance genes removal dynamics in chicken manure by composting. Bioresour. Technol. 410:131309. doi: 10.1016/j.biortech.2024.131309, 39159726

[ref51] Zhao Z. Li P. Zhang M. Feng W. Tang H. Zhang Z. (2024). Unlocking the potential of Chinese herbal medicine residue-derived biochar as an efficient adsorbent for high-performance tetracycline removal. Environ. Res. 252:118425. doi: 10.1016/j.envres.2024.118425, 38325789

[ref52] Zhou S. Li H. Wu Z. Li S. Cao Z. Ma B. . (2023). The addition of nano zero-valent iron during compost maturation effectively removes intracellular and extracellular antibiotic resistance genes by reducing the abundance of potential host bacteria. Bioresour. Technol. 384:129350. doi: 10.1016/j.biortech.2023.129350, 37352990

[ref53] Zhou Y. Selvam A. Wong J. W. C. (2018). Chinese medicinal herbal residues as a bulking agent for food waste composting. Bioresour. Technol. 249, 182–188. doi: 10.1016/j.biortech.2017.09.212, 29040853

[ref54] Zhou Y. Zhang Z. Awasthi M. K. (2022). Exploring the impact of biochar supplement on the dynamics of antibiotic resistant fungi during pig manure composting. Environ. Pollut. 314:120235. doi: 10.1016/j.envpol.2022.120235, 36165829

[ref55] Zhu P. Wu Y. Ru Y. Hou Y. San K. W. Yu X. . (2022). Industrial-scale aerobic composting of livestock manures with the addition of biochar: variation of bacterial community and antibiotic resistance genes caused by various composting stages. Environ. Pollut. 314:120270. doi: 10.1016/j.envpol.2022.120270, 36162559

