# Supplementary material for: Addition of Chinese medicinal herbal residues promotes the removal of antibiotic resistance genes during pig manure composting
Source: Front Microbiol. 2026 Oct 14;17:1932472. doi: 10.3389/fmicb.2026.1932472 (PMC13714935; doi:10.3389/fmicb.2026.1932472)
Supplement: Supplementary file 1 [file Table_1.DOCX]

**Table S1** Basic physical and chemical properties of compost raw materials

| Raw material | pH | EC (mS·cm^-1^) | TOC (g/kg) | TN (g/kg) | Moisture (%) |
| --- | --- | --- | --- | --- | --- |
| pig manure | 7.53±0.14 | 5.22±0.35 | 386.54±13 | 25.42±0.84 | 8.36±0.02 |
| corn stover | 6.84±0.06 | 2.34±0.03 | 425.82±16 | 5.63±0.51 | 5.37±0.04 |
| CMHRs | 5.31±0.02 | 3.23±0.01 | 568.65±8 | 28.36±0.65 | 12.87±0.88 |

The values in parentheses are the mean ± standard deviation (n=3).

TOC, total organic carbon; TN, total nitrogen.

**Table S2** Basic information of the tested CMHRs

| Latin name | Family | Medicinal parts | Main chemical components |
| --- | --- | --- | --- |
| Cistanches Herba | Orobanchaceae | Fleshy stem | Volatile oil, Flavonoids, Alkaloid |
| Aurantii Fructus Immaturus | Rutaceae | Young fruit | Phenylethanoid glycosides, Polysaccharides, Cyclohexene ether terpenes |

**Table S3** The qPCR primers

| **Assay_**  **name** | **Drug Class** | **Forward primer** | **Reverse Primer** |
| --- | --- | --- | --- |
| *AAC(3)-Ia* | Aminoglycoside antibiotic | ACGTTCTGCCAAAGTTTGAG | ACTGCCGGATCGTCAC |
| *AAC(3)-Ib* | Aminoglycoside antibiotic | CAGCGAGACGTTCATCGC | CACGCTTCAGGTGGCTAATC |
| *AAC(3)-Iic* | Aminoglycoside antibiotic | ACGGCATTCTCGATTGCTTT | CCGAGCTTCACGTAAGCATTT |
| *Aac(3)-iid_iia_iie* | Aminoglycoside antibiotic | CGATGGTCGCGGTTGGTC | TCGGCGTAGTGCAATGCG |
| *AAC(3)-IV* | Aminoglycoside antibiotic | CCAACACGACGCTGCATC | GCTGTCGCCACAATGTCG |
| *AAC(3)-Via* | Aminoglycoside antibiotic | GTGTCCGTCGCCAAGGA | GGTGACGGCCTTGTCGA |
| *AAC(3)-Xa* | Aminoglycoside antibiotic | GCAAGCGGTTCGTGACGTA | TCAGGTGCTCCTCGATCCAG |
| *AAC(6')-Ib* | Aminoglycoside antibiotic | CGTCGCCGAGCAACTTG | CGGTACCTTGCCTCTCAAACC |
| *AAC(6')-Ig* | Aminoglycoside antibiotic | GCGATGTTAGAAGCCTCAATTCG | CACACTTCGGCCTGTCGAA |
| *AAC(6')-IIa* | Aminoglycoside antibiotic | CGACCCGACTCCGAACAA | GCACGAATCCTGCCTTCTCA |
| *AAC(6')-IIc* | Aminoglycoside antibiotic | CAGTCTTTGGCTAATCCATCACAG | AACGAACCCGGCCTTCTC |
| *AAC(6')-Ij* | Aminoglycoside antibiotic | ATGCCTGTATCTGAATCCCTGATG | GGCAATCGCTTGTTGAGTATCTG |
| *AAC(6')-Im* | Aminoglycoside antibiotic | CGTGAGCATTATACAGAGCAATGG | CCATTTCCGTTCGTAGATATTGGC |
| *AAC(6')-Ir* | Aminoglycoside antibiotic | GCTATAACGATCAGCAGCAAGC | CGCGATGCATGGCATGAC |
| *AAC(6')-Iw* | Aminoglycoside antibiotic | TGCGTCAGTTACTTACACGAAC | CCTGATGCATTGCATGACTGA |
| *AAC(6')-Iz* | Aminoglycoside antibiotic | TGCGCCATGACTACGTGAAC | GACTGTCCGAAGCCAGTTCG |
| *aacA43* | Aminoglycoside antibiotic | CTTGGCCTACATTAGATTCAGCTC | GCTCTCAATCTTTGATAGGAGCAG |
| *aadA* | Aminoglycoside antibiotic | GTTGTGCACGACGACATCATT | GGCTCGAAGATACCTGCAAGAA |
| *aadA16* | Aminoglycoside antibiotic | ACGGTGGCCTGAAGCC | GAATTGCAGTTCCCGTCTGG |
| *aadA17* | Aminoglycoside antibiotic | TGTACGGCTCCGCAGTG | CACGGAATGATGTCGTCGTG |
| *aadA2* | Aminoglycoside antibiotic | CAATGACATTCTTGCGGGTATC | GACCTACCAAGGCAACGCTATG |
| *aadA21* | Aminoglycoside antibiotic | ACGGCTCCGCAGTGGAT | GGCCACAGTAACCAACAAATCA |
| *aadA5* | Aminoglycoside antibiotic | ATCACGATCTTGCGATTTTGCT | CTGCGGATGGGCCTAGAAG |
| *aadA6* | Aminoglycoside antibiotic | CCATCGAGCGTCATCTGGAA | CCCGTCTGGCCGGATAAC |
| *aadA7* | Aminoglycoside antibiotic | CACTCCGCGCCTTGGA | TGTGGCGGGCTCGAAG |
| *aadA9* | Aminoglycoside antibiotic | CGCGGCAAGCCTATCTTG | CAAATCAGCGACCGCAGACT |
| *ANT(2'')-Ia* | Aminoglycoside antibiotic | CCTGCTTGGTGGGCAGAC | CGGCACGCAAGACCTCAA |
| *ANT(4')* | Aminoglycoside antibiotic | CCGACAACATTTCTACCATCCTT | ACCGAAGCGCTCGTCGTATA |
| *ANT(6)* | Aminoglycoside antibiotic | TACCTTATTGCCCTTGGAAGAGTTA | GGAACTATGTCCCTTTTAATTCTACAATCT |
| *APH(3')-Ib* | Aminoglycoside antibiotic | AACAGGTTTGGGAGGCGATG | CGCAACAAGCCTCTCCTGAA |
| *APH(4)-Ib* | Aminoglycoside antibiotic | GGGAACACCGTGCTCACC | GTTGGTCCCGTGCAGGTC |
| *APH(6)-Ia* | Aminoglycoside antibiotic | CGCTGGGAGCTGAAGAGG | AGCATCGTGCTGCTCTCC |
| *APH(6)-Ic* | Aminoglycoside antibiotic | CACGACAACGTGCTCGAC | CCGTCTTCGGCGAACCA |
| *APH(6)-Id* | Aminoglycoside antibiotic | GCTCGGTCGTGAGAACAATCT | CAATTTCGGTCGCCTGGTAGT |
| *APH(9)-Ib* | Aminoglycoside antibiotic | GCTATGTGCTGGTGGACTGG | GGAACCACTCGACGAACTCG |
| *APH3-III* | Aminoglycoside antibiotic | CAGAAGGCAATGTCATACCACTTG | GACAGCCGCTTAGCCGAA |
| *dfrA1* | Diaminopyrimidine antibiotic | GGAATGGCCCTGATATTCCA | AGTCTTGCGTCCAACCAACAG |
| *dfrA12* | Diaminopyrimidine antibiotic | CCTCTACCGAACCGTCACACA | GCGACAGCGTTGAAACAACTAC |
| *dfrA14* | Diaminopyrimidine antibiotic | CGGATCATGTCATTGTTTCAGG | ATGTTAGAGGCGAAGTCTTGG |
| *dfrA15* | Diaminopyrimidine antibiotic | AGGCCGAAAGACTTTCGAGTC | TCACCTTCTGGCTCAATGTCG |
| *dfrA17* | Diaminopyrimidine antibiotic | CGGGAACGGCCCTGATATTCC | CGTGTTGCGACCGCATACTTTC |
| *dfrA21* | Diaminopyrimidine antibiotic | TTGTTTCAACGCTGTCGCA | GGTTTCGGTTGAGACAAGCTC |
| *dfrA27* | Diaminopyrimidine antibiotic | GCCGCTCAGGATCGGTA | GTCGAGATATGTAGCGTGTCG |
| *mdtH* | Fluoroquinolone antibiotic | ATGCTGGCTGTACAAGTGATG | CACTCCAGCGGGCGATA |
| *qacA_B* | Fluoroquinolone antibiotic | AAGGGCCACTGCATTAGCTG | CCAGTCCAATCATGCCTGCA |
| *QepA_ 1_2* | Fluoroquinolone antibiotic | GGGCATCGCGCTGTTC | GCGCATCGGTGAAGCC |
| *QnrB4* | Fluoroquinolone antibiotic | TCACCACCCGCACCTG | GGATATCTAAATCGCCCAGTTCC |
| *QnrD* | Fluoroquinolone antibiotic | CGCTGGAATGGCACTGTGA | GCTCTCCATCCAACTTCACTCC |
| *Qnrvc1_vc3_vc6* | Fluoroquinolone antibiotic | CTCACATCAGGACTTGCAAGAA | ATGAAGCATCTCGAAGATCAGC |
| *vanA* | Glycopeptide antibiotic | GGGCTGTGAGGTCGGTTG | TTCAGTACAATGCGGCCGTTA |
| *vanB* | Glycopeptide antibiotic | TTGTCGGCGAAGTGGATCA | AGCCTTTTTCCGGCTCGTT |
| *vanC2_vanC3* | Glycopeptide antibiotic | TGACTGTCGGTGCTTGTGA | GATAGAGCAGCTGAGCTTGTTC |
| *vanHB* | Glycopeptide antibiotic | GAGGTTTCCGAGGCGACAA | CTCTCGGCGGCAGTCGTAT |
| *vanRB* | Glycopeptide antibiotic | GCCCTGTCGGATGACGAA | TTACATAGTCGTCTGCCTCTGCAT |
| *vanTC* | Glycopeptide antibiotic | ACAGTTGCCGCTGGTGAAG | CGTGGCTGGTCGATCAAAA |
| *vanTE* | Glycopeptide antibiotic | GTGGTGCCAAGGAAGTTGCT | CGTAGCCACCGCAAAAAAAT |
| *IS1111* | Insertional | GTCTTAAGGTGGGCTGCGTG | CCCCGAATCTCATTGATCAGC |
| *ISSm2-Xanthob* | Insertional | TGGATCGACCGGTTCCAT | GCTGACCGAGCTGTCCATGT |
| *intI1_337old* | Integrase | GCCTTGATGTTACCCGAGAG | GATCGGTCGAATGCGTGT |
| *IncI1_repI1* | MGE | CGAAAGCCGGACGGCAGAA | TCGTCGTTCCGCCAAGTTCGT |
| *IS1247* | MGE | CGGCCGTCACTGACCAA | TCGGCAGGTTGGTGACG |
| *IS6100* | MGE | CGCACCGGCTTGATCAGTA | CTGCCACGCTCAATACCGA |
| *IS630* | MGE | CCGCCACCAGTGTGATGG | TTGGCGCTGACTGGATGC |
| *ISCR1* | MGE | ATGGTTTCATGCGGGTT | CTGAGGGTGTGAGCGAG |
| *ISEcp1* | MGE | CATGCTCTGCGGTCACTTC | GACGCACCTTCTTGATGACC |
| *EreA* | MLSB | GATAATTCTGCTGGCGCACA | GCAGGCGTGGTCACAAC |
| *EreB* | MLSB | TCGTATATGGCGGGCGTAGTA | GGTCCAAGATGGGTGAATGCA |
| *Erm(35)* | MLSB | CCTTCAGTCAGAACCGGCAA | GCTGATTTGACAGTTGGTGGTG |
| *Erm(36)* | MLSB | GGCGGACCGACTTGCAT | TCTGCGTTGACGACGGTTAC |
| *erm(O)* | MLSB | TGATGACGGCTCAGTGG | GTGCACCAGCGCCTGA |
| *ErmE* | MLSB | GTCACGCAGCTGGAGTTCG | CGGTGAAGCACAGCTCGAC |
| *ErmF* | MLSB | CAGCTTTGGTTGAACATTTACGAA | AAATTCCTAAAATCACAACCGACAA |
| *ErmQ* | MLSB | TGAAAGCCATGCGTCTGAC | TTCAGCTGGCAGCTTAAGC |
| *ErmS* | MLSB | GAGTACGCCCGCAAACG | GCGTTCGATCCGGAGGA |
| *lnuF* | MLSB | ATACCGGTCATTTCCACTTGGC | GCATCAGGCTGATGAGGTTCAA |
| *mphA* | MLSB | TCAGCGGGATGATCGACTG | GAGGGCGTAGAGGGCGTA |
| *oleC* | MLSB | CCCGGAGTCGATGTTCGA | GCCGAAGACGTACACGAACAG |
| *pica* | MLSB | GCAATCGAGGCGGTGTTC | TTGCCGCAGCCAATTCA |
| *pikR2* | MLSB | TCGTGGGCCAGGTGAAGA | TTCCCCTTGCCGGTGAA |
| *vatA* | MLSB | ATGAACGGAGCGAATCATCGG | CCATACCGATCCAAACGTCATTTC |
| *vatB* | MLSB | GCAATTGTTGCTGCGAATTCAG | GTGCTGACCAATCCCACCA |
| *cefa_qacelta* | Multidrug | TAGTTGGCGAAGTAATCGCAAC | TGCGATGCCATAACCGATTATG |
| *ceoA* | Multidrug | ATCAACACGGACCAGGACAAG | GGAAAGTCCGCTCACGATGA |
| *emrD* | Multidrug | CTCAGCAGTATGGTGGTAAGCATT | ACCAGGCGCCGAAGAAC |
| *mdtA* | Multidrug | ACAAGCCCAGGGCCAAC | CCTTAATGGTGCCTTCGGTTTC |
| *mdtE* | Multidrug | CGTCGGCGCACTCGTT | TCCAGACGTTGTACGGTAACCA |
| *mdtG* | Multidrug | TTCCAGCCGGTCAGCAA | GACATCTCCCGCGAGTTCG |
| *mel_ 1* | Multidrug | TAATTATCGCAGCAGCTGGTTC | GTTCCCAAACGGAGTATAAGAGTG |
| *mepA* | Multidrug | ATCGGTCGCTCTTCGTTCAC | ATAAATAGGATCGAGCTGCTGGAT |
| *MexB* | Multidrug | CTGGAGATCGACGACGAGAAG | GAAATCGTTGACGTAGCTGGAA |
| *MexE* | Multidrug | GGTCAGCACCGACAAGGTCTAC | AGCTCGACGTACTTGAGGAACAC |
| *mtrD* | Multidrug | CGGAGTCCATCGACCATTTG | ATCGTCGGCAAGGAGAATCA |
| *OprD* | Multidrug | ATGAAGTGGAGCGCCATTG | GGCCACGGCGAACTGA |
| *qacF_H* | Multidrug | TCGCAACATCCGCATTAAAA | ATGGATTTCAGAACCAGAGAAAGAAA |
| *qacH_351* | Multidrug | GTCGGTGTTGCTTATGCAGTCT | CAACCAGGCAATGGCTGTAA |
| *ttgA* | Multidrug | ACGCCAATGCCAAACGATT | GTCACGGCGCAGCTTGA |
| *ttgB* | Multidrug | TCGCCCTGGATGTACACCTT | ACCATTGCCGACATCAACAAC |
| *bacA* | Peptide antibiotic | ATCCGCGGCACCCTGA | CCTGCTTGATGGACTTGATGAAGA |
| *MCR-1. 1* | Peptide antibiotic | CACATCGACGGCGTATTCTG | CAACGAGCATACCGACATCG |
| *catB3* | Phenicol antibiotic | GCACTCGATGCCTTCCAAAA | AGAGCCGATCCAAACGTCAT |
| *catB9* | Phenicol antibiotic | CACCTTATGAAGTGGTCGGTTCA | GTCTGATGAACACAGAGACTGCA |
| *cmlA1* | Phenicol antibiotic | TAGGAAGCATCGGAACGTTGAT | CAGACCGAGCACGACTGTTG |
| *cmlA5* | Phenicol antibiotic | GCGCTCTTCGAGGATTCG | CCGCCCAAGCAGAAGTAGAC |
| *cmlv* | Phenicol antibiotic | GCCCTCATCACCGTCTTCG | GGACGTTGGCGATGGAGAG |
| *cmx* | Phenicol antibiotic | GCGATCGCCATCCTCTGT | TCGACACGGAGCCTTGGT |
| *floR* | Phenicol antibiotic | AACCCGCCCTCTGGATCA | GCCGTCGAGAAGAAGACGAA |
| *trb-C* | Plasmid | CGGYATWCCGSCSACRCTGCG | GCCACCTGYSBGCAGTCMCC |
| *IncP_oriT* | Plasmid-inc | CAGCCTCGCAGAGCAGGAT | CAGCCGGGCAGGATAGGTGAAGT |
| *sul1* | Sulfonamide antibiotic | GCCGATGAGATCAGACGTATTG | CGCATAGCGCTGGGTTTC |
| *sul2* | Sulfonamide antibiotic | TCATCTGCCAAACTCGTCGTTA | GTCAAAGAACGCCGCAATGT |
| *sulA_folP* | Sulfonamide antibiotic | CAGGCTCGTAAATTGATAGCAGAAG | CTTTCCTTGCGAATCGCTTT |
| *16S* | Taxonomic | GGGTTGCGCTCGTTGC | ATGGYTGTCGTCAGCTCGTG |
| *tet(44)* | Tetracycline antibiotic | CTCATGTAGATGCAGGAAAGACG | GTAACTGCTGCCTGAATTGTGA |
| *tet32* | Tetracycline antibiotic | CCATTACTTCGGACAACGGTAGA | CAATCTCTGTGAGGGCATTTAACA |
| *tetA* | Tetracycline antibiotic | CTCACCAGCCTGACCTCGAT | CACGTTGTTATAGAAGCCGCATAG |
| *tetA(P)* | Tetracycline antibiotic | GGAAACCTTAGTTCAGTGACTTGG | CCCATTTAACCACGCACTGAA |
| *tetB* | Tetracycline antibiotic | AGTGCGCTTTGGATGCTGTA | AGCCCCAGTAGCTCCTGTGA |
| *tetD* | Tetracycline antibiotic | AATTGCACTGCCTGCATTGC | GACAGATTGCCAGCAGCAGA |
| *tetG* | Tetracycline antibiotic | TCGCGTTCCTGCTTGCC | CCGCGAGCGACAAACCA |
| *tetH* | Tetracycline antibiotic | TTTGGGTCATCTTACCAGCATTAA | TTGCGCATTATCATCGACAGA |
| *tetL* | Tetracycline antibiotic | ATGGTTGTAGTTGCGCGCTATAT | ATCGCTGGACCGACTCCTT |
| *tetM* | Tetracycline antibiotic | GGAGCGATTACAGAATTAGGAAGC | TCCATATGTCCTGGCGTGTC |
| *tetO* | Tetracycline antibiotic | CAACATTAACGGAAAGTTTATTGTAT | TTGACGCTCCAAATTCATTGTATC |
| *tetQ* | Tetracycline antibiotic | CGCCTCAGAAGTAAGTTCATACACT | TCGTTCATGCGGATATTATCAGAAT |
| *tetR* | Tetracycline antibiotic | CCGTCAATGCGCTGATGAC | GCCAATCCATCGACAATCACC |
| *tetS* | Tetracycline antibiotic | TTAAGGACAAACTTTCTGACGACAT | TGTCTCCCATTGTTCTGGTTCA |
| *tetT* | Tetracycline antibiotic | CCATATAGAGGTTCCACCAAATCC | TGACCCTATTGGTAGTGGTTCTATTG |
| *tetW* | Tetracycline antibiotic | ATGAACATTCCCACCGTTATCTTT | ATATCGGCGGAGAGCTTATCC |
| *tetX* | Tetracycline antibiotic | AAATTTGTTACCGACACGGAAGTT | CATAGCTGAAAAAATCCAGGACAGTT |
| *tnpA-2* | Transposase | CCGATCACGGAAAGCTCAAG | GGCTCGCATGACTTCGAATC |
| *tnpA-3* | Transposase | GGGCGGGTCGATTGAAA | GTGGGCGGGATCTGCTT |
| *ACT beta-lac* | β-Lactamase | AAGCCGCTCAAGCTGGA | GCCATATCCTGCACGTTGG |
| *blaSFO* | β-Lactamase | CCGCCGCCATCCAGTA | GGGCCGCCAAGATGCT |
| *CARB beta-lac* | β-Lactamase | TGATTTGAGGGATACGACAACTCC | CTGTAATACTCCGAGCACCAA |
| *CcrA beta-lac* | β-Lactamase | CACTGGCACGGCGATTGTA | CGGCAGCCAAACCACGATA |
| *class C beta-lac* | β-Lactamase | CTGGCGCATACCTGGATTAC | GCCAGTTCAGCATCTCCCA |
| *CphA beta-lac* | β-Lactamase | GCGAGCTGCACAAGCTGAT | CGGCCCAGTCGCTCTTC |
| *CTX-M-1_3_ 15* | β-Lactamase | CGTACCGAGCCGACGTTAA | CAACCCAGGAAGCAGGCA |
| *MIR beta-lac* | β-Lactamase | CGGTCTGCCGTTACAGGTG | AAAGACCCGCGTCGTCATG |
| *OXY-1-1* | β-Lactamase | AAAGGTGACCGCATTCGC | CCAGCGTCAGCTTGCG |
| *OXY-2-1* | β-Lactamase | CGTTCAGGCGGCAGGTT | GCCGCGATATAAGATTTGAGAATT |
| *penA* | β-Lactamase | AGACGGTAACGTATAACTTTTTGAA | GCGTGTAGCCGGCAATG |
| *SHV-11* | β-Lactamase | TTGACCGCTGGGAAACGG | TCCGGTCTTATCGGCGATAAAC |
| *VIM beta-lac* | β-Lactamase | GCACTTCTCGCGGAGATTG | CGACGGTGATGCGTACGTT |
